# Diffusion Barriers Minimizing the Strength Degradation of Reactive Air Brazed Ba_0.5_Sr_0.5_Co_0.8_Fe_0.2_O_3-δ_ Membranes during Aging

**DOI:** 10.3390/membranes13050504

**Published:** 2023-05-10

**Authors:** Simone Herzog, Anke Kaletsch, Christoph Broeckmann

**Affiliations:** Institute for Materials Applications in Mechanical Engineering, RWTH Aachen University, Augustinerbach 4, 52062 Aachen, Germany

**Keywords:** four-point bending joint strength, reactive air brazing, BSCF, oxygen transport membrane, isothermal aging, diffusion barrier

## Abstract

The separation of oxygen from air by means of inorganic ceramic membranes requires gas-tight ceramic–metal joints that enable reliable permeation operation in the oxygen partial pressure gradient at 850 °C. Reactive air brazing is a promising method to solve this challenge. However, reactive air brazed BSCF membranes suffer from a significant strength degradation that is caused by unhindered diffusion from the metal component during aging. In this study, we investigated how diffusion layers applied on the austenitic steel AISI 314 influence the bending strength of BSCF-Ag3CuO-AISI314 joints after aging. Three different approaches were compared as diffusion barriers: (1) aluminizing via pack cementation, (2) spray coating with NiCoCrAlReY, and (3) spray coating with NiCoCrAlReY and an additional 7YSZ top layer. Coated steel components were brazed to bending bars and aged for 1000 h at 850 °C in air prior to four-point bending and subsequent macroscopic as well microscopic analyses. In particular, coating with NiCoCrAlReY showed low-defect microstructures. The characteristic joint strength was raised from 17 MPa to 35 MPa after 1000 h aging at 850 °C. In addition, the dominant delamination fracture between the steel and the mixed oxide layer, observed in the reference series with uncoated steel, could be replaced by mixed and ceramic fractures of higher strength. The effect of residual joint stresses on the crack formation and path is analyzed and discussed. Chromium poisoning could no longer be detected in the BSCF, and interdiffusion through the braze was effectively reduced. Since the strength degradation of reactive air brazed joints is mainly caused by the metallic joining partner, the findings on the effect of the diffusion barriers in BSCF joints might be transferred to numerous other joining systems.

## 1. Introduction

Oxygen supply by means of oxygen transport membranes (OTMs) can contribute to CO_2_ avoidance to fulfill CCS strategies (carbon capture and storage). Demand for oxygen separated from the air exists in (raw) material production, medical applications, the chemical industry, and water treatment, in addition to the efficient combustion of fossil or renewable fuels. It is important that the oxygen enrichment or separation equipment itself can operate safely and energy efficiently at typical process temperatures of about 850 °C. Oxygen output is maximized for thin membranes, high gradients of oxygen partial pressure on both sides of the membrane, high operating temperatures, and membrane materials with high non-stoichiometry δ. As result of a high non-stoichiometry, the combined coefficient of thermal and chemical expansion increases, as in the case of the membrane material Ba_0.5_Sr_0.5_Co_0.8_Fe_0.2_O_3-δ_ (BSCF) [1,2,3,4].

**Figure 1:**
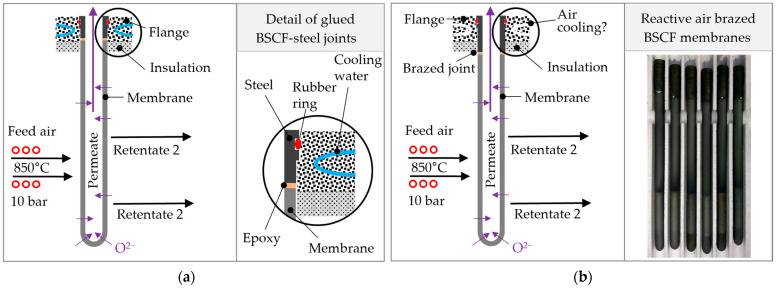
Comparison of module concepts for tubular OTMs: (**a**) ceramic membrane glued with adhesives to metal sleeves with rubber rings and mounted in a water-cooled flange and (**b**) ceramic membranes brazed to metal sleeves and exemplary photograph of reactive air brazed membrane tubes.

OTM concepts therefore vary in the selected membrane materials, the geometry of the membrane component, the process gases, and their partial pressures but also the sealing concepts. Demonstrator modules often work with tubular membranes that are glued with adhesives to metal sleeves [5,6,7], as schematically also shown in Figure 1a. Due to the water cooling in the flange and the insulation to the heated pressure vessel, the temperature-sensitive adhesives and rubber sealing rings remain cool. However, an axial temperature gradient occurs along the length of the membrane.

Observed BSCF membrane fractures in a previous study suggest that this axial temperature gradient leads to a local stress maximum as a result of the different minimum temperatures for the onset of chemical expansion or creep relaxation [8]. In tubular membranes; however, it is, compared with planar membrane stacks, relatively easy to shift the axial temperature gradient to the metallic joining partner. For this purpose, the permanent cooled adhesive joint between the membrane tube and the metal sleeve used in the membrane module must be replaced by a gas-tight and high-temperature-resistant joint; see Figure 1b. In addition to increasing the reliability, this can increase the efficiency as a result of an increase in the effective membrane area contributing to permeation and heat losses due to water cooling.

To join fragile BSCF membrane tubes to metals in a gas-tight and high-temperature-resistant manner, only brazing methods can be considered. For permeation studies of thin membrane discs at the laboratory scale, press-fit joints with gold gaskets and spring elements are often used [9,10]. However, these compression joints are not suitable for small-scale joining of long, free-hanging membrane tubes due to boundary conditions. As early as 1994, Sarocco et al. identified the joining technology as a key problem for the application of OTMs as membrane reactors, which five years later was said to have received too little attention and progress [11,12].

Among the brazing techniques, some established technologies exist to realize ceramic–metal joints [13]. However, this is more difficult due to the strong ionic or ionic-covalent bonds in ceramics. Moreover, the processes of vacuum brazing, active brazing, or brazing of a metallized ceramic are not suitable, because BSCF chemically decomposes under low-oxygen partial pressures or through reducing reactions with the reactive elements [14,15]. In addition, these braze alloys or the locally reduced ceramic oxidize during subsequent application under air, which causes volume expansion and leads to cracks and delamination [15,16,17]. Glass or glass–ceramic solders, as used for joints in solid oxide fuel cells stacks [18,19], are currently not suitable for BSCF joints. Their low thermal expansion coefficients in relation to BSCF in combination with the brittle fracture lead to high internal stresses and cracks upon cooling [20], which can be detected, e.g., by N_2_ contents in the permeate [21]. There are approaches to increase the coefficient of thermal expansion of glass solders by targeted crystallization of oxide phases. Approaches exist to increase the thermal expansion coefficient of glass solders by selective crystallization of oxide phases [22]. Chemical interactions can be reduced by gold coating of the membrane [23], but thermal cycling also changes the degree of crystallization and the thermomechanical properties [24], which can damage the solder and ceramic membrane.

Reactive brazing in air has been shown to be suitable for joining BSCF to high-temperature-resistant metals [25,26,27,28]. In addition, other OTM materials such as LSCF [29,30], BCFN [31], or dual-phase membranes [32,33,34] were joined using reactive air brazing. Suitable metallic joining partners show a similar thermal expansion to the ceramic OTM. Examples include ferritic steels such as Kanthal [35], Crofer APU or H [36], AISI 430 [37], AISI 446 [38], austenitic heat-resistant steels such as AISI 310S [33], AISI 314 [39], and nickel-base alloys such as alloy 602 CA [40], Haynes 214 [35], or Inconel 600 [33].

The BSCF membrane tubes shown in Figure 1b are brazed to steel sleeves in this manner using Ag-3Cu (3 mol% Cu, 97 mol% Ag). A similar concept with Ag-1Cu was shown by Zhang et al. [31,41]. During the brazing process in air, the reactive element copper is oxidized and wets reactively both the oxide ceramic and the passivated metal surface in the silver melt. However, during membrane operation, the progressive diffusion of copper, chromium, and oxygen leads to the growth of the reaction layers, which strongly decreases the strength of the joints [42,43,44]. In particular, the diffusion of chromium from the metallic joining partner leads to microstructural decomposition [35,42,43] and a decrease in permeation performance [45].

Some coatings have been tested to limit chromium diffusion in brazed joints and show stabilized microstructures. The Ni-based alloy 602 CA was coated with an Al_2_O_3_ top layer using the reactive air aluminizing technique [46] and then air brazed. Strength studies after aging have not been published. In another study [47], AISI 314 was coated with Ni-Al_2_O_3_ and YSZ via thermal spraying and then brazed in air using pure silver. After 1500 h of Al aging at 850 °C, no growing reaction layers were observed in cross-sections. In [39], we demonstrated how a pre-oxidation of AISI 314 at 1050 °C can reduce the strength degradation of BSCF-Ag-3CuO-AISI 314 joints after 1000 h of isothermal aging at 850 °C. Without pre-oxidation, the characteristic joint strength decreases from 81 MPa immediately after brazing to 17 MPa after 1000 h, and chromium poisoning is present. With pre-oxidation before brazing, 33 MPa joint strength is achieved, and no chromium is detected in the BSCF.

Dense outer Al_2_O_3_ passivation layers could be even better diffusion barriers. They are only formed in materials with sufficient Al reservoirs that are >6 wt%. However, there are no heat-resistant austenitic steels with sufficiently high Al content [48], since these would form intermetallic phases during cooling. The choice of a ferritic high-Al steel led to cracking due to the large differences in thermal expansion coefficients [39]. In the present study, therefore, aluminum-containing coatings or diffusion layers were applied onto the metallic joining partner before brazing to BSCF and aging. Four-point bending tests, fracture surface analyses, and categorization as well as microstructure analyses of cross-sections enable the discussion of the efficacy of the applied coatings as a diffusion barrier for air brazed BSCF–steel joints.

## 2. Materials and Methods

Ba_0,5_Sr_0,5_Co_0,8_Fe_0,2_O_3-δ_ powder (Treibacher GmbH, Althofen, Austria) with an average particle size of 3 µm was transformed into granules appropriate for pressing by spray drying (Dorst Technologies GmbH, Kochel am See, Germany, 125 µm average granule size). Cylindric bars were pressed in two stages to realize the desired high aspect ratio h/d ≈ 1.3. An amount of 2.8 mg granules was pressed in a double-acting die at 50 MPa on a manual hydraulic uniaxial press (PW40, Paul-Otto We-ber, Remshalden, Germany) to cylindrical green bodies with a diameter of 9.5 mm. After vacuuming in foil, cold isostatic pressing (EPSI at 180 MPa for 60 s) was carried out. Sintering of the rods was conducted in a chamber furnace (HT64/16, Nabertherm, Lilienthal, Germany) at 1100 °C for 5 h in air atmosphere.

Metallic braze foil with an average composition of 97 mol% silver and 3 mol% copper was produced by electroplating 75 µm thick silver foils with 1.6 µm copper layers on both sides. The braze foil was punched out with a diameter of 8 mm, cleaned, and smoothed. During the brazing process, the oxidation of the reactive element copper under the formation of composition Ag-3CuO took place.

Bars made of the heat-resistant austenitic steel AISI 314 (DIN X15CrNiSi25-21, EN 1.4841) were coated with different variants. As a reference condition, we refer to results published in [39] where blank AISI 314 was brazed to BSCF without any diffusion barrier. Aluminizing was carried out by solid-state diffusion in the pack cementation process at Chromin Maastricht BV, Maastricht, The Netherlands. Aluminized metal components were oxidized in air at 1050 °C either for 1 h or for 100 h to improve the wetting of the braze. NiCoCrAlReY layers were applied by high-velocity flame spraying (HVOF) at the Institute for Surface Technology in Aachen (series H). The same institute carried out an additional coating with 7 mol% yttria-stabilized zirconia (7YSZ) by atmospheric plasma spraying (APS) on some samples with NiCoCrAlReY coating. Table 1 gives an overview of the abbreviations used.

The brazing of the joints was conducted after the vertical arrangement of all components in a specially constructed brazing frame; see Figure 1a,b. Details regarding the brazing frame are given in [39]. The programmed temperature–time regime of a brazing cycle is given in Table 2.

After brazing, the isothermal aging was conducted at 850 °C for 1000 h (Ecotop 20, Rohde, München, Germany). To avoid stress cracks, the maximum heating and cooling rate here was limited to 150 K/h.

The aged joints were tested at room temperature in accordance with the four-point bending test of design B standardized in DIN EN 843-1 for monolithic ceramics. The specimen lay symmetrically on the support rollers with a distance of L = 40 mm and thus both joint areas within the span of l = 20 mm between the two load rollers. After applying a pre-load of 1 N, the centrally placed joints were loaded until failure in a universal testing machine (Z020, Zwick/Roell, Ulm, Germany). The fracture stress $\sigma_{i}$

(1) $$\sigma_{i}=\frac{8F_{max}(L-l)}{\pi d^{3}}$$

is calculated with the measured maximum force $F_{max}$ and the joint diameter d = 8.1 mm.

**Figure 2:**
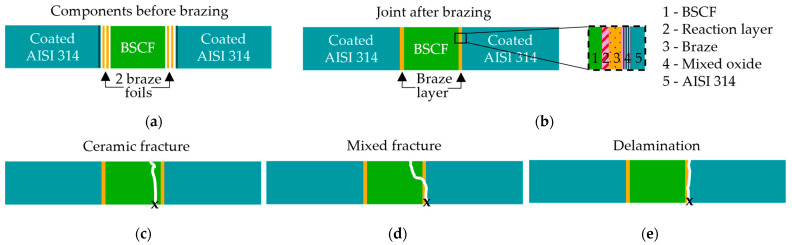
Arrangement of the components (**a**) before and (**b**) after brazing under formation of five different typical interlayers between BSCF and AISI314. (**c**–**e**) Illustrations of the three categories of fracture types where the “x” marks the fracture origin.

Representative cross-sections were made of specimens with a bending strength close to the average strength of the series. Overview images in the light microscope (Axioscope 7, Zeiss, Jena, Germany) gave an overview of typical defects in the joints. This was followed by higher-magnification analyses in a scanning electron microscope (FEI Helios Nanolab G3 CX DualBeam, Thermofisher, Waltham, MA, USA). The fracture surfaces of all joints were assigned to the three fracture types, “ceramic fracture”, “mixed fracture”, and “delamination”, after images were taken under the stereo microscope (KL 1500LCD, Olympus, Tokyo, Japan). Figure 2c,d illustrate the representative crack paths of each fracture types where “x” marks the fracture origin at the tensile loaded position.

## 3. Results

### 3.1. Defect Formation

**Figure 3:**
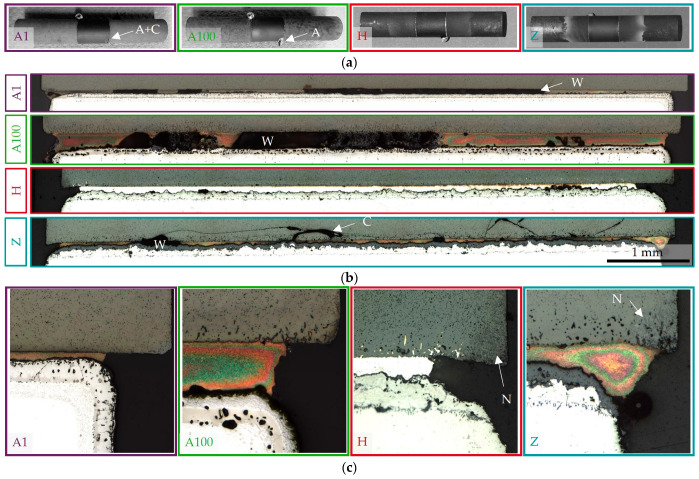
Illustration of different defects per series visible on the macro- and meso-scale. (**a**) Photos highlighting adhesion of braze drops (A) on the brazing frame and (A + C) adhesion followed by chipping in the BSCF. (**b**) Overview of the entire joint cross-sections. Arrangement in cross-sections: top—BSCF; bottom—AISI 314. W indicates wetting gaps, and C indicates cracks. (**c**) Optical microscope images of one edge of the joints. The white arrow indicates a needle-like BSCF structure (N).

After brazing, one or two braze drops were observed; see Figure 3a. When two braze drops were formed, they were distributed over the two joining zones. The fracture propagation was not affected by the positioning of braze drops on the side or on the top surface subjected to compressive stress. However, some braze drops adhered to the brazing frame, which is why affected specimens required light blows with the rubber mallet through the inspection opening before removal (see A1 and A100). This occasionally resulted in small chipping (see A1). We explained the clustered adhesion of the solder drops by the preferential wetting of the solder on the pre-oxidized brazing frame made of X15CrNiSi15-12 instead of the aluminized and 1 h pre-oxidized steel.

**Figure 9:**
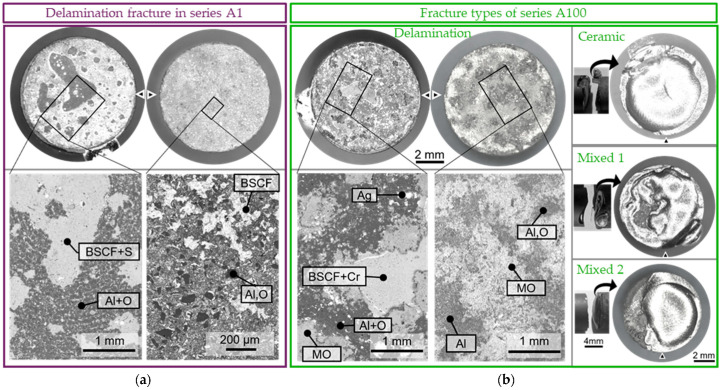
(**a**) Typical delamination fracture surfaces of series A1 and (**b**) typical fracture surfaces of series A100. Details are BSE images with EDX results; MO describes a mixed oxide of the cations Al/Cr/Mn/Ni/Ba/Sr/Co/Fe.

The optical microscope images in Figure 3b give an overview of the entire joint zone of the representative sections. Note that no statistically relevant number of joints was prepared. However, the large wetting gaps (W) in the samples from series A1 and A100 are remarkable and correlate with large pores on fracture surfaces of these series which will be later described in Figure 9a,b. Furthermore, numerous round gas pores were observed in in the diffusion coating and the transition to the basic microstructure of the AISI 314 steel in aluminized samples. The size of the pores increased with the duration of the pre-oxidation from A1 to A100. While the wetting in Series H and Z was homogenous, large crack networks were observed in Series H. These cracks were supposed to origin at small wetting gaps where local stress concentration occurred. Cracks, but with a completely different crack progression, have already been observed in a previous study in BSCF-Ag-3CuO-Kanthal joints [39]. It cannot be proven when the cracks were formed. In the case of the 7YSZ top layer in series Z and the ferritic steel Kanthal [39], however, high residual stresses arising during cooling from the brazing temperature are not unexpected. According to the simulations in [43], they are four times higher in BSCF–Crofer22H joints than in BSCF–AISI314 joints. The differing crack occurrence could be explained by the difference in thermal expansion coefficients at brazing temperature; see Table 3. In series Z, the stresses led to breakout of entire BSCF fragments and explained the strikingly multifaceted and partly curved ceramic or mixed fractures, which will be discussed in Section 3.4.

Changes in the BSCF microstructure due to the formation of needle-shaped precipitations (N) were present in series Z and H; see Figure 3c. No needles were observed the aluminized series A1 and A100. Remarkably, braze infiltration into the BSCF and pore coarsening of the membrane material was also absent in A1.

### 3.2. Microstructure Analysis

**Figure 4:**
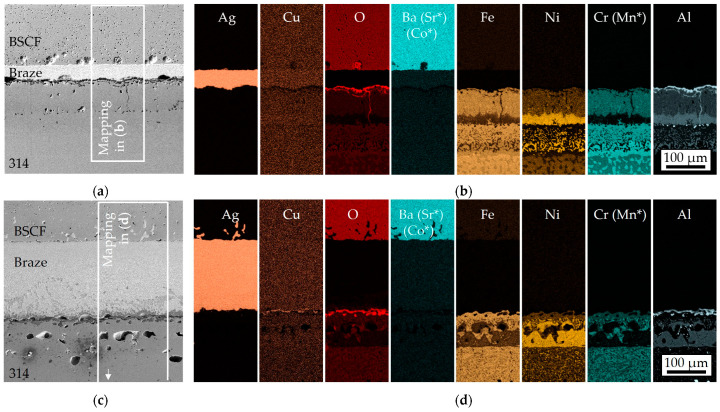
SE images (**a**,**c**) and elemental distribution (**b**,**d**) in series A1 (**top** panel) and A100 (**bottom** panel). Element mappings marked with * are identical to the displayed element mapping.

In joints with aluminized steel, smaller signs of damage were observed in the SEM images. Figure 4a shows a microcrack in the SE contrast of a specimen from series A1, starting at the outer surface layer of the steel, as well as pore accumulations at inner boundary layers in the aluminized steel. In addition, chipping can be seen on the ceramic-side reaction layer, which occurred during preparation. This indicates high stresses and the presence of hard particle abrasion. After 100 h of pre-oxidation, the pores in Figure 4c were significantly coarser, and the internal boundary layers in the steel were no longer visible. The micro-cracks in the diffusion zone of A1 no longer occurred.

The mappings of the element distribution in Figure 4b,d explain the different diffusion layers formed in joints with aluminized steel. After 1 h pre-oxidation, copper was distributed below the reliable EDX detection limit and not locally enriched, as in the reference series. However, after 100 h of pre-oxidation, copper was concentrated in the Al/O top layer. Copper might accumulate in the steel-side mixed oxide layer only if it was already pronounced during brazing. This was consistent with the observations for 1 and 100 h pre-oxidized AISI 314 in [39]. Elements from the BSCF were not involved in the formation of the mixed oxide layer, and conversely, no chromium could be detected in the ceramic. The aluminum enrichment extended about 160 µm deep into the steel component after 1 h of pre-oxidation and was no longer terminated in the image section shown after 100 h of pre-oxidation. The intermetallic Fe_x_Al_y_ phases formed below the Al_2_O_3_ top layer were in principle strongly dependent on the temperature during pack cementation [45,49]. NiAl and Cr_2_Al precipitates also occurred at high aluminum contents in austenitic steels [50,51]. Diffusion annealing caused aluminum cations to penetrate the substrate at higher diffusion rates than iron cations could diffuse to the interface [52]. The resulting iron vacancies coagulated to form pores at the interfaces and were the crack-initiating defects in mechanically stressed components [53].

**Figure 5:**
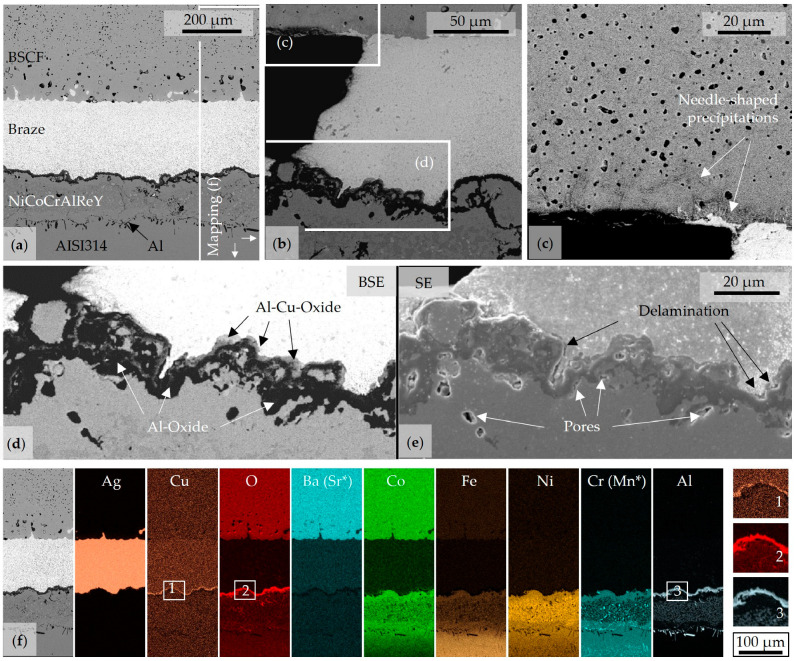
Microstructural characteristics of Series H. (**a**–**c**) Overview and detail BSE images. (**d**,**e**) Same detail in SE and BSE images. (**f**) Mapping of element distribution with details 1–3. Element mappings marked with * are identical to the displayed element mapping.

The steel components of series H joints were sandblasted before coating with NiCoCrAlReY to improve the coating adhesion. The transition between steel substrate and coating was clearly characterized by mainly pure aluminum after joint aging (Figure 5a,f). Since rather wavy interfaces were observed in the literature after HVOF coating [54], microcracks, as observed in [55], may have been healed by diffusion as a result of aging. The braze wetted both BSCF and the formed mixed oxide layer without any noticeable defects. However, large wetting gaps occurred frequently (Figure 5b). At these braze–BSCF–air triple points, needle-like precipitates in the BSCF were observed at high magnification in Figure 5c. Comparative BSE and SE images indicate first damage at the braze–metal interface; see Figure 5d,e. Both pores and an oxide appear black in the BSE contrast and have to be differentiated via SE contrast and EDX mapping in Figure 5f. Accordingly, dark-appearing Al oxide completely covered the metal surface, while a light-appearing Al-Cu oxide did not occur continuously between the braze and the copper-free Al-oxide. At positions where the Al-Cu-oxide was interrupted, pores or already locally delaminated areas were frequently identified. The light Al/Cu-oxide presumably had an adhesion-promoting effect. According to the element mapping, the dark oxide was a Cu-free Al_2_O_3_. The layer immediately below was depleted of aluminum (see details 1–3), so the pores between the metal and dark oxide in Figure 5d were probably Kirkendall pores. Below, a heterogeneous aluminum-rich layer, which probably contained the typical β-aluminum reservoir (see Figure S6, Supplementary Materials), and the Al inclusions followed. The latter consisted of pure aluminum and could not be identified as detrimental to the bond strength. Overall, the gradients in the metal substrate were less sharp than in the aluminized joints. The presence of a continuous thin aluminum oxide layer proved that pre-oxidation of the NiCoCrAlReY coated steel substrates was not necessary. In contrast to the reference series in [39], the strong interdiffusion of chromium and barium/strontium was significantly reduced.

**Figure 6:**
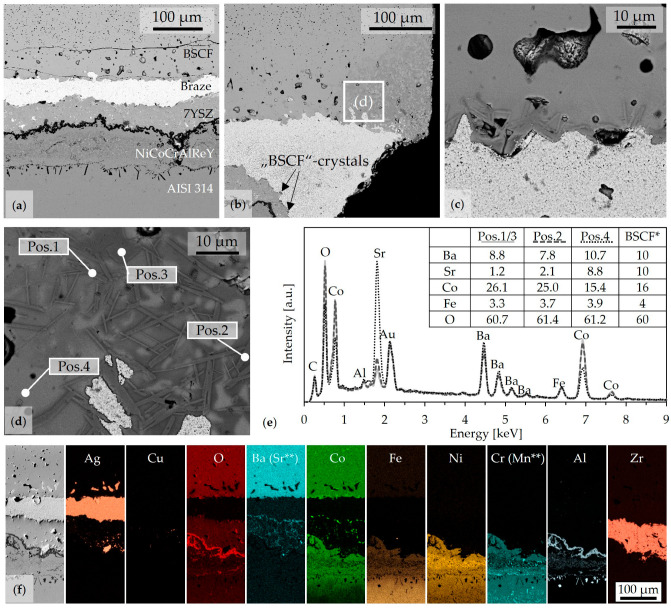
Microstructural characteristics of Series Z. (**a**–**d**) BSE images with marked EDX positions, (**e**) EDX spectra with quantitative element concentration in mol% after correction (Au sputtering layer, C contamination, and Al values corrected). * Ideal BSCF composition. (**f**) Mapping of element distribution. Element mappings marked with ** are identical to the displayed element mapping.

In series Z, an additional 7YSZ layer was applied by atmospheric plasma spraying on top of the NiCoCrAlReY layer. The anomalies in the element distribution previously described for series H were consistent for series Z; see Figure 6f. Chromium was not detected in the ceramic. Since 7YSZ had very good ionic conductivity, oxygen ions from the air or braze were probably transported to the interface coated with NiCoCrAlReY. Therefore, a pronounced inner Al_2_O_3_ layer also appeared in joints of series Z. The 7YSZ layer appeared porous in the mapping and was locally infiltrated by silver and enriched with the elements barium, strontium, and cobalt from the BSCF. This was presumably a composition similar to the BSCF whereby iron with only 4 at% was not detectable in the ideal perovskite. The particles similar to BSCF are also readily visible in the BSE image in Figure 6a,b as distinct crystals at the braze-7YSZ interface. Incipient delamination or accumulation of pores was not observed at this interface. Instead, the cracks in the BSCF known from the light microscopy overview images in Figure 3b were confirmed as critical defects in the joint. The cracks ran predominantly parallel to the braze surface and passed pores. The crack origin could not be identified. It was possible that these cracks were grown subcritically due to the load during the bending test. In micrographs, the “intact” joint was examined in each case after aging and bending testing. 

Despite any detected diffusion of chromium, needle-shaped precipitates were visible in the BSCF directly above the braze (Figure 6c) or in the edge region of the joint (Figure 6d). The formation of the precipitates was accompanied by decomposition of the BSCF. Both needles (Pos.1 and Pos.2) and dark gray decomposed BSCF matrix (Pos.3) showed significantly reduced strontium contents with simultaneously increased cobalt contents, whereas the light gray BSCF matrix (Pos.4) with the quantitative measured values was relatively close to the ideal perovskite (BSCF*).

### 3.3. Fracture Strength

Not all the 82 brazed joints listed in Table 4 could be tested by four-point bending. Some joints in Series H and Z adhered to the brazing frame and were damaged during removal. Three joints of Series A1 delaminated already during aging, as also described in [42]. However, most of the joints (94%) could be aged and tested.

**Figure 7:**
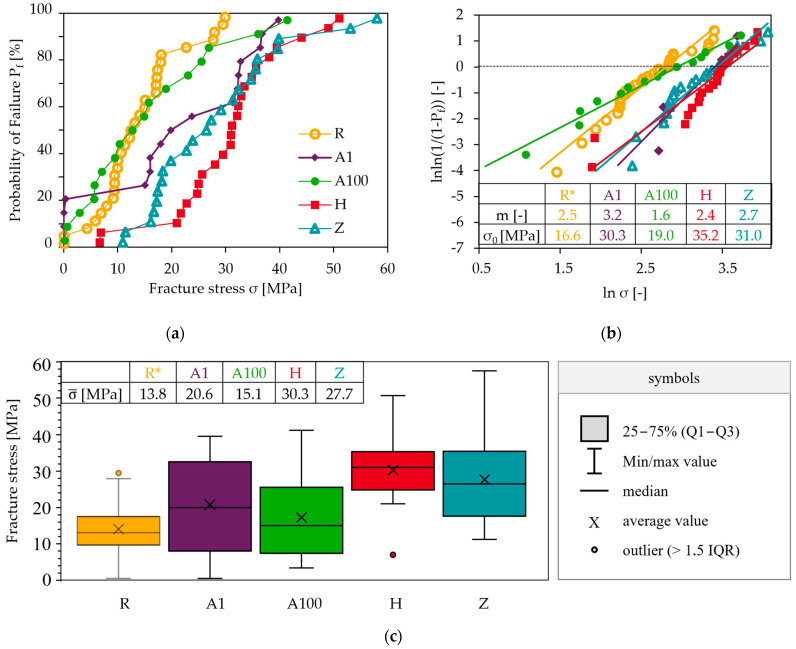
Results of the four-point bending of the joints after 1000 h aging at 850 °C. (**a**) Probability of failure vs. fracture stress. The probability of failure is calculated using the estimation function P_f,i_ = (i − 0.5)/N with the rank of the *i*-th sample and the total number of tested samples N. (**b**) Weibull plot with Weibull parameters given in the inset. (**c**) Boxplots of the fracture stress with median strength $\bar{\sigma }$ in the inset, fractures outside the 1.5 IQR (interquartile distance, length of box) are marked separately. * Data of the reference series R from [39] are shown for comparison.

Measured and calculated strength test data are available in [56] (document F). When the probability of failure was plotted against the fracture stress, a sigmoidal function was expected. However, Table 4 already shows some specimens that failed prematurely (at “0 N”) or at fracture forces below 10 N. This led to the discontinuous curve in Figure 7a. For the Weibull evaluation in Figure 7b, these data points had to be excluded, which may have distorted the result. However, the procedure could also be interpreted as a synthetic proof test. In comparison with the reference series, all diffusion barriers tested in Series A1, H, and Z doubled the characteristic strength σ_0._ The Weibull modulus, represented by the slope in Figure 7b, remained in a similar range. This was unexpected, especially due to the high crack density in series Z described before.

In addition to Weibull evaluations, the determined fracture stresses were evaluated by means of box plots; see Figure 7c. This allowed the inclusion of the strength of specimens that failed during aging or at very low fracture forces. The median value $\bar{\sigma }$ could be understood complementary to the characteristic strength of the Weibull evaluation. Graphically, Figure 7c illustrates the advantageous and very narrow distribution of series H, the premature fracture of joints in series R and A1, but also the highest achieved fracture stresses in series Z.

### 3.4. Fracture Surfaces and Types

Each pair of generated fracture surfaces was differentiated into ceramic fracture, mixed fracture, and delamination fracture categories using stereo microscope images. Finally, this allowed a correlation of the fracture stresses with fracture types. Overall, no simultaneous fracture was observed at both joint positions in any of the specimens tested. Furthermore, the steadily increasing force–displacement curve and the subsequent optical analysis gave no indications for damage of the second joint position. This means the weakest link theory used for the evaluation in Section 3.3 was valid.

**Figure 8:**
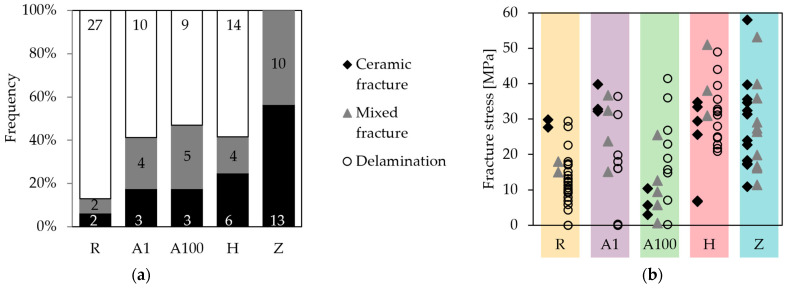
(**a**) Percentage of fracture types in the respective sample series. The numbers in the pillars indicate the absolute number. (**b**) Correlation and distribution of the determined fracture stresses with the assigned fracture types within the different sample series. Data of the reference Series R were obtained from [39].

The relative and absolute frequencies of the fracture types in the respective specimen series are shown in Figure 8a. While the delamination fracture clearly dominated in the reference series with 87%, this fracture type occurred less frequently in Series A1, A100, and H. In Series Z, delamination fractures did not occur at all. The plot in Figure 8b depicts the fracture stress values of all samples categorized in the three fracture type categories. The mean strength of the delamination fracture in the reference series was significantly increased in Series H. In Series A100, the ceramic and mixed fractures exhibited a poor strength.

In the aluminized joints, 59 and 53% (A1 and A100, respectively) failed as a plane delamination fracture. The exposed surfaces were often similarly light grey in color, and the braze layer always remained completely on the long fragment containing the BSCF component. Figure 9a shows an exemplary fracture pattern of series A1 with a very large pore on the ceramic half (left). The mirror-image surface of the pore was not visible on the short fragment (right).

In some delaminated samples, such as the selected fracture pattern of series A100 in Figure 9b, the surfaces appeared heterogeneous, and pores could be recognized to some extent on the short fragment. In general, the wetting was better on 100 h pre-oxidized, aluminized steel than on only 1 h pre-oxidized, aluminized steel since in series A1, pores are observed on the ceramic fragment in all 10 delamination fractures and in series A100 only in two of nine delamination fractures.

To track in which plane the fracture occurred, Figure 9 shows SEM details with EDX analyses. Elements of BSCF and sulfur were detected at large wetting gaps of series A1 (Figure 9a, left). The sulfur could, as discussed by Kaletsch et al. on the basis of formation enthalpies [42], originate from traces in the steel and preferentially form (Ba,Sr)SO_4_. Aluminum oxide was exposed on the turning grooves, which remained even after aluminizing and pre-oxidation. In higher magnification of the short fragment (Figure 9a, right), pure alumina was detected for the dark grey, angular crystals and BSCF for the light grey areas. In medium grey, a heterogeneous mixture of BSCF and aluminum appeared. After 100 h pre-oxidation, aluminum oxide was also present on both fracture surfaces; see Figure 9b. In addition to BSCF, chromium was also present on wetting gaps. The silver dots on the large fragment (shown on the left) indicate infiltration of the mixed oxide layer. The mixed oxide (MO) appeared light grey and consisted of alternating proportions of oxygen, aluminum, chromium, iron, and elements of the BSCF. At some positions on the small fragment (right), almost metallic aluminum was present. The delamination fracture was thus predominantly within an aluminum oxide layer on series A1. Since the braze remained completely on the large fragment (both shown on the left), the BSCF particles reached the mixed oxide layer through the braze. In series A100, the fracture occurred between the mixed oxide layer, the aluminum oxide layer, and the aluminized base material.

The ceramic and mixed fractures of the aluminized joints differ to those observed in the reference series where mainly faceted fracture surfaces were observed [39]. While in series A1, exclusively plane fractures occurred in the ceramic, these were curved to wavy after 100 h pre-oxidation. Figure 9b (right panel) shows an exemplary ceramic fracture of series A100 where the crack occurred as a circumferential normal stress fracture relatively close to the joining zone. After about one millimeter, the fracture surface curved into the large fragment and again approached a relatively flat fracture surface in the inner area. The fracture pattern was similar in the mixed fractures, where delaminated areas were exposed in the center of the specimen or on one side. This fracture behavior could not be reconciled with the typical stress profile in four-point bending and must therefore be strongly dominated by internal defects and residual stresses.

**Figure 10:**
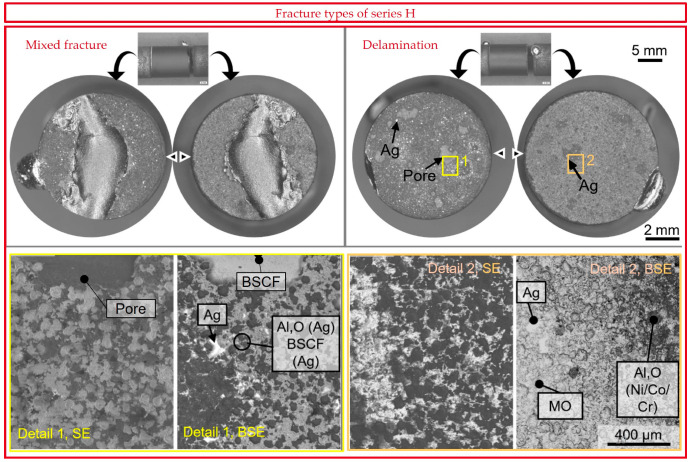
Typical mixed fracture surfaces and delamination surfaces of series H. Details 1 and 2 of the shown delamination fracture as SE and BSE images with EDX results are given below. MO describes a mixed oxide of the cations Al/Cr/Mn/Ni/Ba/Sr/Co/Fe.

**Figure 11:**
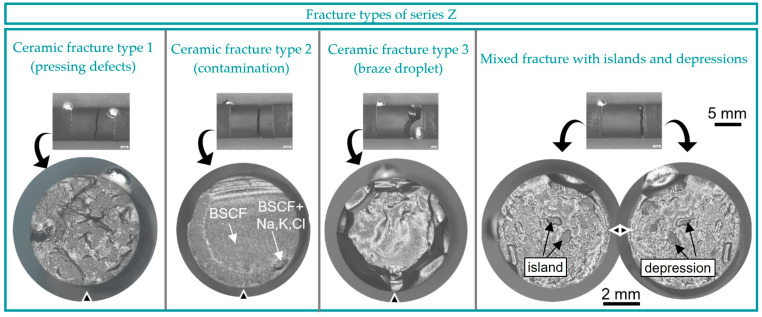
Typical ceramic fracture surfaces with three different subtypes and mixed fracture with peaks and valleys of series Z.

The H series specimens were coated with the NiCoCrAlReY and directly brazed in this condition. Delamination fractures occurred in 58% of all joints. Mixed fractures started exclusively as delamination fractures and ended mostly as curved ceramic fractures. Figure 10 shows the only sample in which the ceramic fracture in the compressive stress range led back between two delaminating layers. The ceramic fractures were similar to those of the Z series shown later in Figure 11.

In delaminated joints of the H series, pores were visible on the long fragment, which were opposed by a darker mixed oxide layer with silver droplets in mirror image on the long fragment. Even when braze droplets adhered to the short fragment, as shown in Figure 10 (delamination), the complete braze layer was covered by a mixed oxide layer on the long fragment. The detailed magnification 2, as presented on the bottom right in Figure 10, demonstrates the typical surface topography of an HVOF-coated surface on the short fragment as described in [57]. Flake-like deposited and partially or non-molten particles can be seen, partially covered with Al-(Ni/Co/Cr) mixed oxides. The “particle imprints” are visible on the surface of the long fragment (detail 1, bottom left), with Al_2_O_3_ present in the cavities (dark in the BSE image) and elements of BSCF on the flatter surfaces. The delamination fracture in series H thus ran partly inside and partly below the alumina layer.

In addition to the NiCoCrAlReY layer, an oxide ceramic top layer of 7YSZ was applied in series Z. After 1000 h of aging, no delamination fractures occurred, and 57% of the joints failed purely in the ceramic and 43% as mixed fracture. Critical defects of the ceramic fractures were well visible and differentiated in three types. For example, Figure 11 shows a rough fracture surface with pressing defects, which were visible as granule boundaries in the scanning electron microscope (Supplementary Materials, Figure S1). The pressing defects occurred only in fractures through the center of the BSCF component. There, the local compaction pressure was low compared with the top surface due to friction [58]. In addition, locally contaminated areas with the elements Na, Ka, and Cl were detected in type 2. Type 3 of ceramic fractures was influenced by the stress field of the braze drops and was faceted on the outside and curved on the inside.

The mixed fracture in Figure 11 was always a straight normal stress fracture initiated at max. 1 mm distance to the braze layer. Here, similar to series A100 in Figure 9b, the fracture surface was often curved with local islands or depressions. The surface of these islands was flat and presumably a locally non-wetted BSCF surface. This matched the observation of the depressions, which appeared dark gray and were wetted with braze drops.

## 4. Discussion

In thin-walled tubular membrane joints with 920 µm wall thickness and contact to the gaseous atmosphere on both sides, distances in the order of magnitude of the wall thickness can be bridged by diffusion after only a short aging time [59]. In addition to the strength-determining defects, special attention must be paid to the microstructural changes at the edges and center of the BSCF. Table 5 compares these as numbered microstructural features of the braze and the BSCF to discuss possible causal relationships between individual observed microstructural changes.

In the previous work, we discussed why chromium is probably transported to BSCF via internal Ba-Cr oxides in the braze in the uncoated Series R. The diffusion barrier studies now performed seem to confirm this, as neither the Ba-Cr oxide phases nor chromium poisoning of the BSCF ⑥ were detected by EDX.

**Figure 12:**
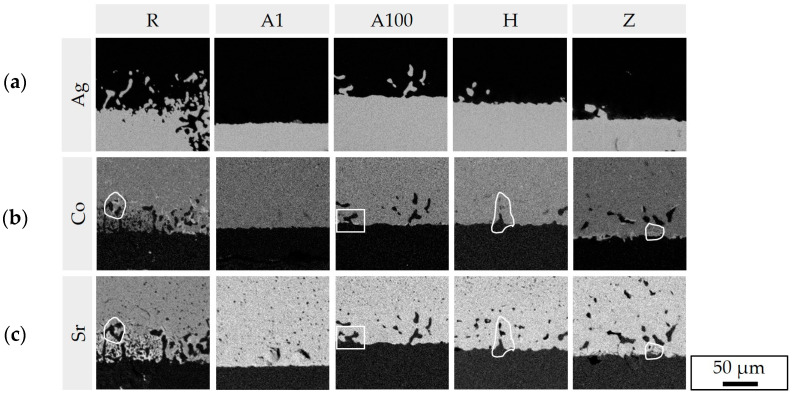
Local element distribution at the interface braze/BSCF: (**a**) silver infiltration into BSCF ① and (**b**,**c**) local Co enrichment and Sr depletion at the braze/BSCF interface ⑤. Encircled numbers refer to microstructural characteristics in Table 5.

The different braze infiltration ① is clearly illustrated by the Ag mappings in Figure 12. In joints (series R) with strong braze infiltration, no pore coarsening occurred in the BSCF ③. This is in agreement with observations in [42], where infiltration without pore coarsening occurred in BSCF-Ag-xCuO-X15CrNiSi25-21 joints, but pore coarsening without infiltration occurred in the wetting test BSCF-Ag-xCuO. The mechanisms of pore coarsening due to locally increased grain boundary mobility have been described in [60], but it is unclear when it occurs.

Braze infiltration did not directly correlate with the local Cu concentration in the mixed oxide layer ②. However, it was noticeable that braze infiltration was pronounced when either many elements of the BSCF diffused through the braze to the metal-side mixed oxide layer ⑦ or the copper was concentrated at this layer ②. For series R, O1, and O99+, a continuous Co-Cu mixed oxide was formed, and for series K, a thick layer of (Al)-Ba-Co-Cr-Fe-Sr mixed oxide was deposited on the metal-side Al_2_O_3_ top layer [39]. Weak infiltration was observed in series A100, H, and Z, where a thin Al-Cu mixed oxide was observed (A100, H), and few distinct Ba-Sr-Co-(Fe) crystallites were deposited on the zirconia layer (Z). In series A1, the pure Al_2_O_3_ top layer was present at the interface to the braze, and no infiltration occurred at all. A possible explanation is the following:

If elements of the BSCF ⑦ or copper ② diffused from the braze to the metal-side interface, voids were created. These were filled either by silver diffusing in or by BSCF (= pore coarsening ③). This would mean that silver could enter between the BSCF grains both primarily during brazing and secondarily by solid-state diffusion during aging. Proof of this hypothesis should be clarified by subsequent studies. This could also be the reason why “infiltration structures” were observed preferentially at wetting gaps and edges, as illustrated, for example, by the overview image of the joints from series A100 in Figure 3b. Continuous braze layers with as few wetting gaps as possible were obviously important not only for beneficial stress transfer and resulting strength but also for microstructural stability.

The occurrence of needle-shaped phases ④ correlated with the observation of decomposition features of the BSCF ⑤. By EDX in Figure 6e, the decomposition was described as enrichment of cobalt with simultaneous depletion of strontium both in the needles and in the surrounding BSCF. The local decomposition of the BSCF was attributed to chromium and sulfur poisoning in [1]. The results now available show that decomposition starts even in the absence of chromium ⑥ and sulfur contamination. If elemental mappings of cobalt and strontium of the same microstructural sections are compared in Figure 12b,c, this tendency can be traced for all sample series except series A1.

The inhomogeneity in the Co and Sr mappings correlated with the formation of needle-like phases (R, H and Z series). Where only differences in the contour of defined structures between Co and Sr mapping were apparent (outlined areas in Figure 12), needles were either weak or absent. However, it can be assumed that the concentration differences of cobalt and strontium in these joints will increase in prolonged aging, and thus, the precipitation of the acicular structures is also thermodynamically favored. Since no Co/Sr inhomogeneity occurred after 1000 h aging of the A1 series, it is possible that the formation of needles will also be absent in long-term use.

A phase analysis of the needles was difficult since only EDX measurements were available where the excitation volume exceeded the volume of the needles. The decomposition of the cubic phase was indicated by the high Goldschmidt factor of 1.07, which clearly exceeded the tolerance range of 0.8–1 for the cubic perovskite structure. This factor was calculated based on the Ba:Sr and Co:Fe cation ratios determined by EDX (Figure 6e). Similar lamellar or plate-like structures were studied in [61,62,63,64] using TEM lamellae, XRD, and dilatometry. The authors describe Co enrichment and Sr depletion in a trigonal phase Ba_3_Co_10_O_17_B with high iron solubility. Depending on the p_O2_-T history, the lamellar trigonal phase was often surrounded by hexagonal phase. Müller observed nucleation at CoO precipitates and a volume fraction of 8% after annealing at 850 °C for 10 h [61]. Decomposition of the BSCF into the iron-free hexagonal Ba(Sr)CoO_3-δ_ phase and cobalt-free cubic Sr(Ba)FeO_3-δ_ phase, as observed in [65] by TEM for the hexagonal and cubic phases, was apparently not present. Moreover, the hexagonal phase showed a different morphology and occurred preferentially at grain boundaries, where it appeared bright in the BSE image [8].

The morphology of the needles in the reactive air brazed BSCF, as well as their protrusion from the cross-section plane due to a locally higher hardness, agreed very well with the descriptions of the trigonal phase. According to EDX analyses in Figure 6e, an ion ratio A:B:O of 10:30:60 was present. This was between the 12.5:25:62.5 [64] and 10:33:56 [62] ion ratios reported in the literature. Instead of nucleation at CoO precipitates, as observed by Müller, the phase boundaries to the braze or the joint surface could favor nucleation. Since the formation of the needles did not occur in the joints with aluminized AISI314 (the same in Series K in [39]), the formation of the trigonal phase in brazed joints cannot depend purely on the p_O2_-T history. The inhomogeneity in the Co-Sr mapping can probably be considered as an early-warning indicator to the formation of visible needles. The lack of braze infiltration ① and resulting low and flat interface to the BSCF with few nucleation sites could be a reason why the A1 series was also free of any decomposition features in the Co and Sr mapping ⑤.

For the geometry of the tested brazed joints, the largest defects in the micrograph and fracture types with the lowest strength from the fracture surface analysis were additional important criteria for discussion. In addition, the percentage of specimens that failed below a 10 MPa proof test limit that was reasonable for the application was relevant to the fabrication route. Table 6 compares these characteristics.

In the R, O1, and O99+ series of our previous study [39], local delamination or elongated pores between the mixed oxide layer and the steel were the largest defects in the micrograph and also the critical defects that initiated delamination. In addition, the observed cracks in series Z triggered the ceramic fracture or a mixed fracture initiated in the ceramic. However, in the series A1, A100, and H, the defects in the micrograph did not determine the fracture type with the lowest strength. The common approach in the literature of correlating joint strength with microstructural defects can lead to erroneous conclusions. Table 7 summarizes the fracture types with the characteristic fracture patterns, defects, and average strength.

While in most series, the average strength of ceramic fractures was above that of mixed fracture and delamination fracture, this order was changed in series H. BSCF was the weakest link in these joints, and the adhesion of steel and mixed oxide layer was advantageous. The strength of the mixed fracture, which started in the mixed oxide layer but passed through the braze in the stress neutral center, was still higher probably due to the crack deflection and enlarged fracture work. An unused strength potential lay obviously in the ceramic component. Since no severe defects were observed in the BSCF and the crack progression was atypical for a flexural test, residual stresses seemed to be responsible for the ceramic fracture type.

These residual stresses were also responsible for the pre-damage caused by microcracks parallel to the braze layer in series Z. In these joints, crack propagation was influenced both by the residual stresses in the coated metal substrate and by the thermally induced stresses during the cooling of the joint. It was therefore surprising that in series H and Z, the ceramic fractures were similar. The series differed in the additional 7YSZ top layer applied in series Z. The 8YSZ layer had a comparatively low coefficient of thermal expansion in the range 12∙10^−6^ K^−1^, similar to that of ferritic steels used in series K [39]. Nevertheless, the pre-damage due to axial cracking in series K and lateral cracking in series Z was completely different. The reasons for this again lie in the residual stress states, which have not yet been investigated and which, in addition to the coefficients of thermal expansion, are strongly influenced by the layer thickness or length of the components.

For the application of the tubular air brazed membrane in a membrane module outlined in Figure 1b, the strengths achieved are theoretically sufficient. In addition to the thermally induced residual stresses, the joint will be loaded with compressive stresses in operation equal to the feed pressure or, in the depressurized state before/after operation, stresses of about 30 kPa due to the dead weight of the membrane itself. Important for the application is the significant increase in the observed minimum strength to avoid failure of the brazed joint during operation. The minimum strength was increased from 0 MPa in joints with uncoated AISI 314 to 7 and 11 MPa by applying the NiCoCrAlReY coating and NiCoCrAlReY layer with 7YSZ top layer, respectively.

## 5. Conclusions

In this work, four different coatings of the metallic joining partner were investigated for their effectiveness as diffusion barriers in reactive brazed and aged BSCF joints. The main findings were as follows:

- Aluminizing inhibited chromium diffusion and the growth of mixed oxide layers, but the formed Al_2_O_3_ top layer was poorly wetted by the braze. The joint strength after isothermal aging could be improved more efficiently by other coatings.
- Coating with NiCoCrAlReY led to well-wetted interfaces, prevented chromium poisoning of the ceramic, and as critical defects only small pores were observed at the mixed oxide layer.
- These small pores could promote delamination at low strengths after growth during extended aging periods. So far, there is no experience on the growth kinetics of these pores nor experiments showing the influence of pre-oxidation of the coated steel components.
- The average strength of the dominant delamination fracture observed in unprotected AISI314 (reference series) could be raised from 12 to 31 MPa by coating the AISI 314 with NiCoCrAlReY. An additional 7YSZ top layer resulted in no delamination fractures at all.
- Differences between Co and Sr mapping of the BSCF in vicinity to the braze were interpreted as an early-warning indicator of BSCF decomposition.

## Figures and Tables

**Table 1:** Series with associated pre-treatment of the metallic joining partners.

Series	Condition of Metallic Component Before Brazing	Number of Brazed Joints
R ^1^	AISI 314, blank without any diffusion barrier ^1^	33 ^1^
A1	AISI 314, aluminized and pre-oxidized for 1 h at 1050 °C	17
A100	AISI 314, aluminized and pre-oxidized for 100 h at 1050 °C	15
H	AISI 314, sand-blasted, HVOF-coated with NiCoCrAlReY	25
Z	AISI 314, sand-blasted, HVOF-coated with NiCoCrAlReY, APS coated with 7YSZ	25

^1^ Results of reference series published in [39].

**Table 2:** Temperature–time profile of the brazing process.

Segment	Rate [K/h]	Temperature	Holding Time [min]
1	300	870	---
2	150	970	6
3	150	600	60
4	150	RT	---

**Table 3:** Difference of thermal expansion coefficients (RT-950 °C) between BSCF and the surface of the joining partner in ppm/K. Data were obtained from typical material data sheets of suppliers.

	Crofer22H	AISI 314	Kanthal APM^®^	7YSZ
BSCF	7	1	4	9

**Table 4:** Number of rejects and reason.

	R *	A1	A100	H	Z
Brazed	33 *	17	15	25	25
Rejected after brazing	2 *	---	---	1	2
Rejected after aging	2 *	3	---	---	---
Fracture force <10 N	--- *	1	2	---	---

* Data of the reference series R from [39] are shown for comparison.

**Table 5:** Comparison of microstructural characteristics.

	R *	A1	A100	H	Z
① Braze infiltration into BSCF	✓	✕	◯	◯	◯
② Local copper concentration in MO	✕	✕	✓	✓	◯
③ Pore coarsening in BSCF	✕	✕	✕	✓	✓
④ Needle-shaped phases in BSCF	✓	✕	✕	✓	✓
⑤BSCF decomposition (Co/Sr)	✓	✕	◯	◯	✓
⑥ Chromium detected in BSCF	✓	✕	✕	✕	✕
⑦ Elements of BSCF pass the braze	✓	✕	✕	✕	✓

Symbols: ✓—applies, ✕—applies not, ◯—applies rarely or weakly true. * Data of the reference series R from [39] are shown for comparison.

**Table 6:** Summary of critical defects in micrographs and fracture types.

	R *	A1	A100	H	Z
Largest defect in microstructure [-]	D	W	W/P	P	Cr
Fracture type with the lowest strength [-]	D	D	C	C	C/M
Lowest strength [MPa]	0	0	0	7	11
Fraction of failures below 10 MPa [%]	31	7	47	8	0

Abbreviations: D—delamination; W—wetting gap; P—pore; Cr—cracks; C—ceramic fracture; M—mixed fracture. * Data of the reference series R from [39] are shown for comparison.

**Table 7:** Comparison of typical fracture patterns and their frequency and average strengths. Subtypes a) and b) are indicated where applicable. The crack path is marked in black. Fracture types with the lowest strength are marked in bold.

	Ceramic Fracture		Mixed Fracture		Delamination	
Series A1	18%	35 ± 4 MPa	23%	27 ± 10 MPa	59%	14 ± 13 MPa
	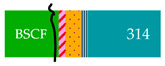		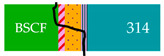		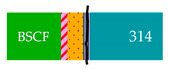	
Series A100	18%	6 ± 4 MPa	29%	11 ± 9 MPa	53%	20 ± 13 MPa
	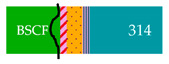		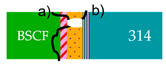		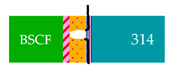	
Series H	25%	23 ± 13 MPa	17%	38 ± 9 MPa	58%	31 ± 8 MPa
	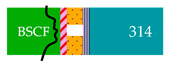		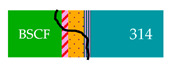		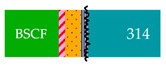	
Series Z	57%	28 ± 13 MPa	43%	28 ± 13 MPa		
	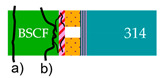		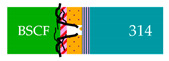		---	

Color Code: 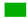 BSCF; 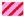 Reaction Layer; 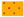 Braze; 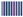 Mixed oxide; 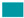 AISI 314.

## Data Availability

Additional data are given in the Supplementary Materials. Datasets are published in https://doi.org/10.18154/RWTH-2022-06816, accessed on 5 May 2023 [56].

## Supplementary Materials

The following supporting information can be downloaded at https://www.mdpi.com/article/10.3390/membranes13050504/s1: Figure S1: SE image shown as exemplary ceramic fracture type 1 of series Z in Figure 7. The initial granules with d50 = 125 µm are visible. This fracture type 1 occurs mainly in the center of the ceramic sample where the pressure during the uniaxial pre-compaction of the BSCF granules is lowest. Figure S2: Area EDX measurement on the fracture surface in Figure 1. No chromium or other impurities were detected. Figure S3: SE image shown as exemplary ceramic fracture type 2 of series Z in Figure 7. The point EDX analyses reveal no chromium poisoning, but local Na, K, and Cl contamination is given in Figure 4 and Figure 5. Figure S4: EDX1, position marked in Figure 3. Figure S5: EDX2, position marked in Figure 3. Figure S6: XRD phase analysis on aluminized and 1 h pre-oxidized AISI314 and on NiCoCrAlReY coated AISI314.
