# Supplementary material for: Diffusion Barriers Minimizing the Strength Degradation of Reactive Air Brazed Ba0.5Sr0.5Co0.8Fe0.2O3-δ Membranes during Aging
Source: Membranes (Basel). 2023 May 10;13(5):504. doi: 10.3390/membranes13050504 (PMC10221055; doi:10.3390/membranes13050504)
Supplement: Supplementary file 1 [file membranes-13-00504-s001.zip › membranes-2356912-supplementary.pdf]

Supplemental Material

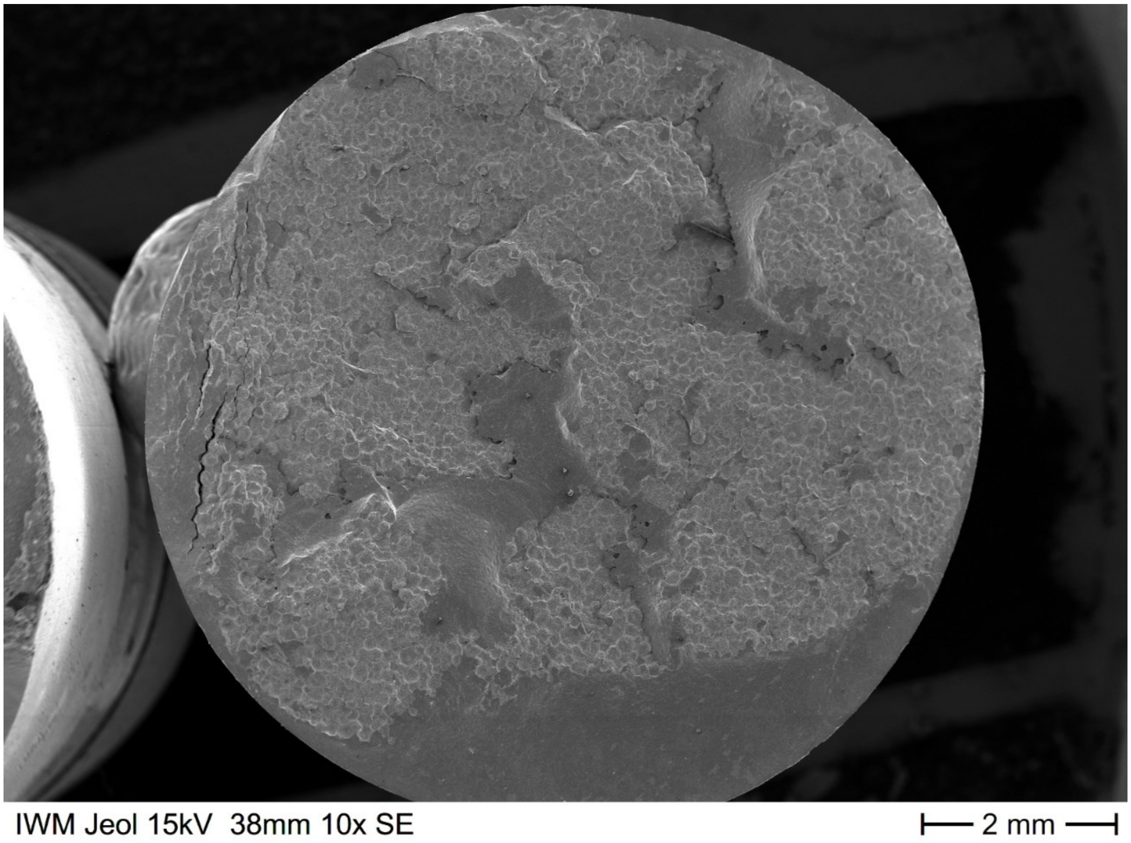

Figure S1: SE image shown as exemplary ceramic fracture type 1 of series Z in Figure 7. The initial granules with d50 = 125 µm are visible. This fracture type 1 occurs mainly in the center of the ceramic sample were the pressure during the uniaxial pre-compaction of the BSCF granules is lowest.

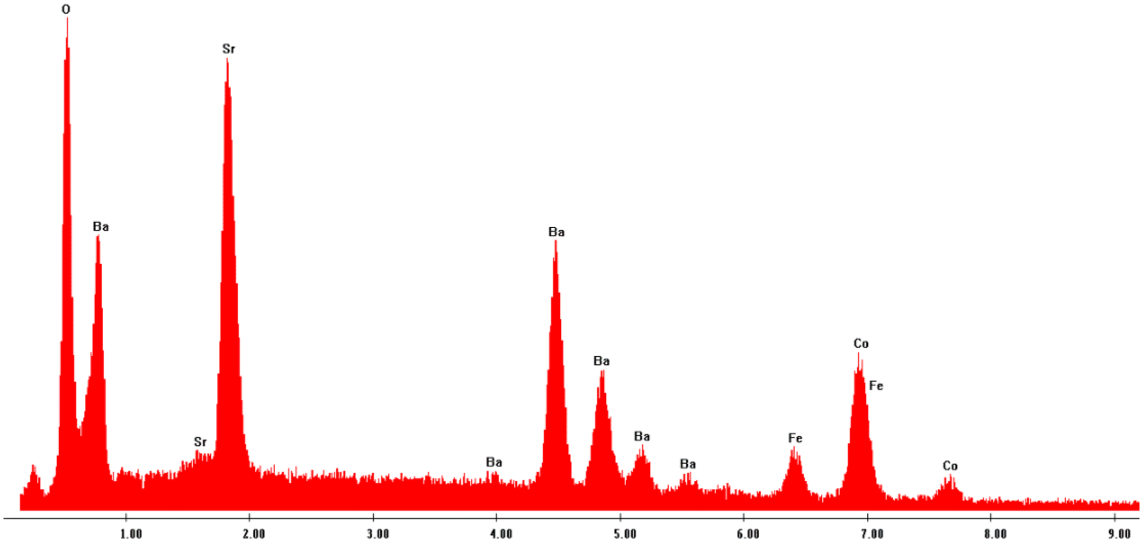

Figure S2: Area EDX measurement on the fracture surface in Figure 1. No chromium or other impurities were detected.

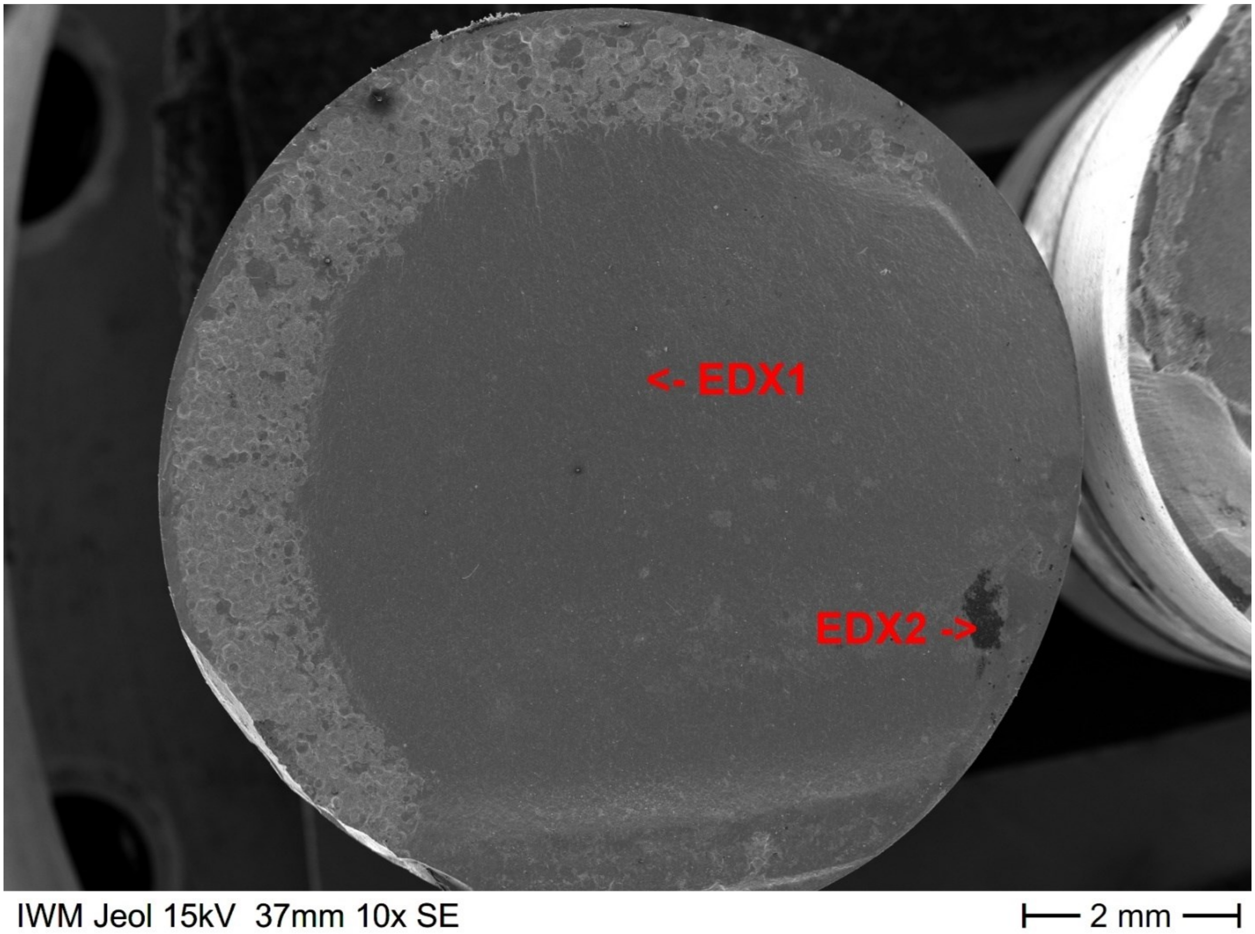

Figure S3: SE image shown as exemplary ceramic fracture type 2 of series Z in Figure 7. The point EDX analyses revealing no chromium poisoning, but local Na, K, Cl contamination are given in Figure 4 and 5, respectively.

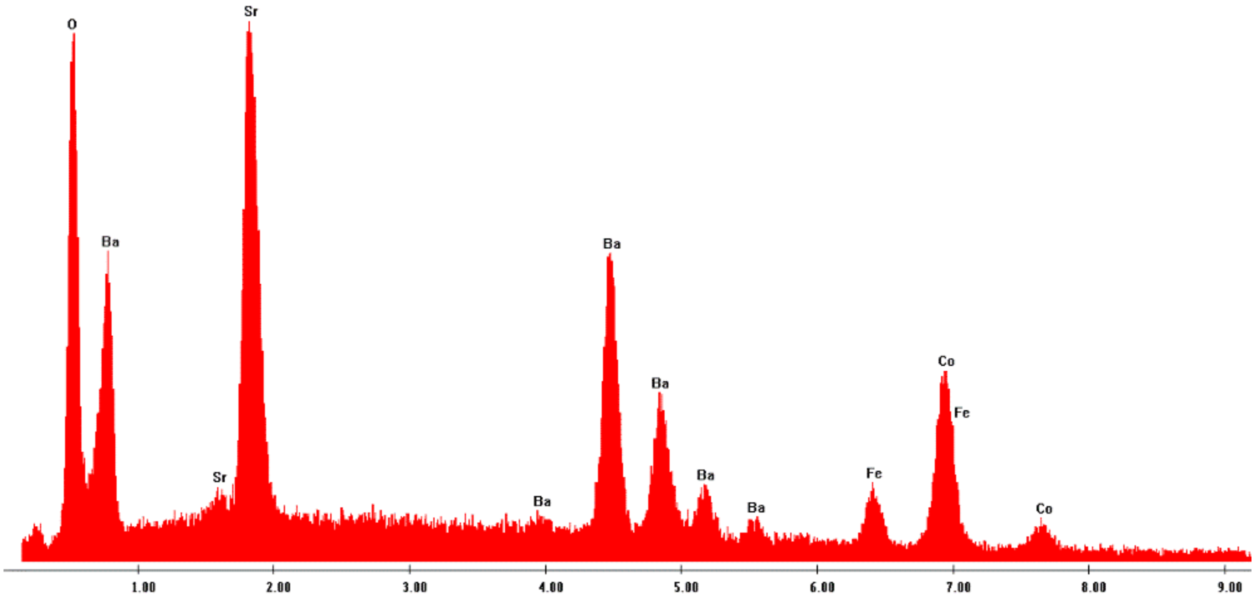

Figure S4: EDX1, position marked in Figure 3

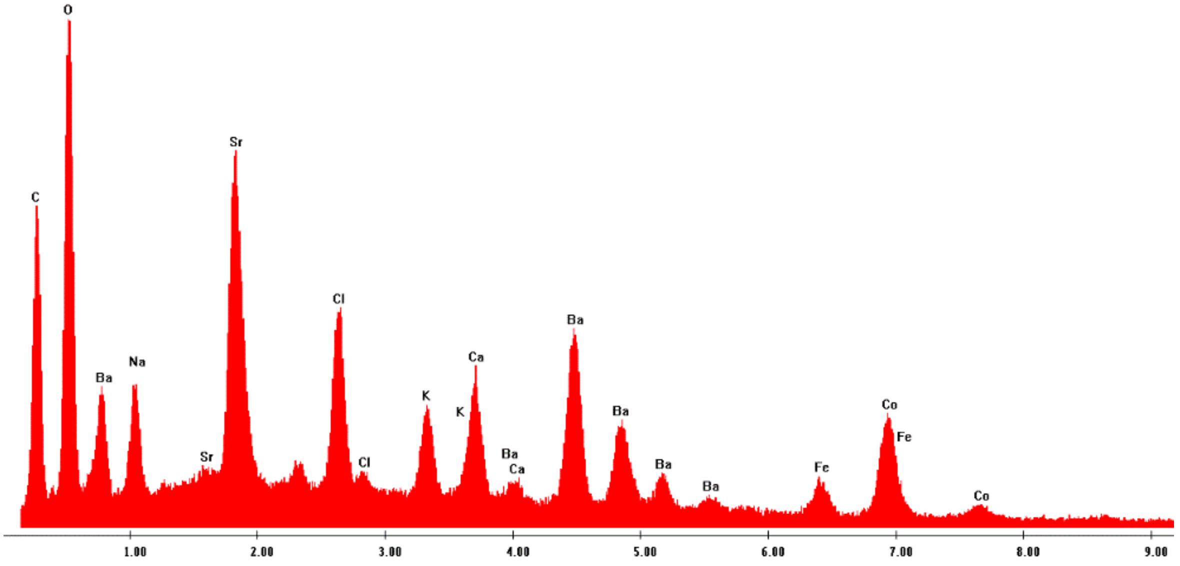

Figure S5: EDX2, position marked in Figure 3

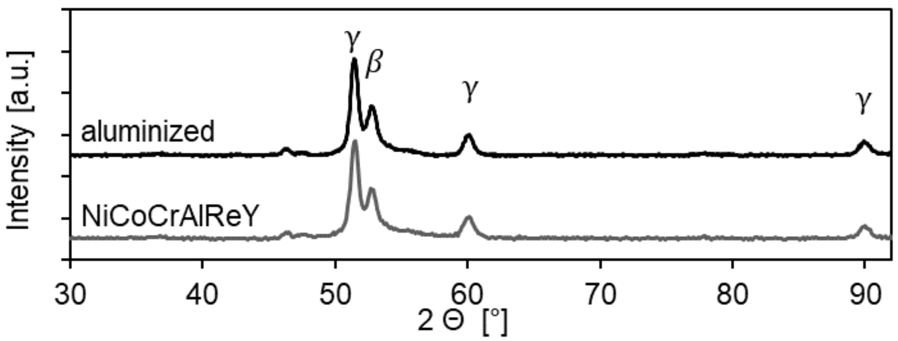

Figure S6: XRD phase analysis on aluminized and 1 h pre-oxidized AISI314 and on NiCoCrAlReY coated AISI314.
